# Two Interesting Cases of Pneumonia

**Published:** 1819-10

**Authors:** Ed. Sutliffe


FOR THE LONDON MEDICAL AND PHYSICAL JOURNAL.

*Two interesting Cases of Pneumonia.* By ED. SUTLIFFE, Esq.

IN the month of March last, Miss B. of Hoxton, was seized with rigor, fever, pain in the chest, difficulty of breathing, &c. When medical aid was called in, the usual depletory remedies were employed: considerable relief was obtained by blistering, purging, digitalis, &c. but the fullness of the pulse seemed to authorize the use of the lancet. About twelve ounces of blood were removed. The character of the blood justified the evacuation; but the patient appeared to labour under an instantaneous increase of all the symptoms. The question now necessarily became serious and important: as the pulse continued full, what measures were to be pursued? In this dilemma I was requested to see the party. We agreed in consultation, that, although the pulse was full and bounding, justifying *in toto* all the measures hitherto pursued, no further relief was to be expected by venesection, fearing subsequently phthisis pulmonalis. We had the pleasure to witness the gradual recovery of this delicate female of æt. 19; but with indubitable symptoms of hectic, which, by care in diet, and by breathing her native air in Hoxton, subsided.

I have no hesitation in affirming, without the fear of contradiction, that this subject would have inevitably fallen into phthisis pulmonalis, had the venesection been repeated two or three times more. Again,

"Aude alteram partem."

Miss H. of Nicholas-lane, æt. 21, from exposure to cold three months ago, laboured under fever, ushered-in by rigors, difficulty of breathing, cough, &c. Although the subject was by no means athletic, the fullness of the pulse at once determined the propriety of venesection; the relief from it was instantaneous: the quantity removed was about sixteen ounces. It is scarcely needful to add, that diuretic drinks constituted her whole support; and that blistering the chest, purgatives, digitalis, &c. were added. However, the cough returning, with a full pulse, a second bleeding to about the same amount was employed, and with the same advantage. In three or four days more, however, it was found necessary, from the recurrence of the diseased symptoms, to repeat venesection in about the same quantity even to the sixth time, (the blood invariably exhibiting criteria of highly-marked arterial excitement,) when steadiness of the pulse, and return of health, began to appear; and convalescence slowly supervened. During this train of formidable symptoms, much pus was coughed-up, with mucus of a strongly saline taste, and occasionally streaked with blood. The sputa were copious and purulent.

The parents and anxious relatives of both these young ladies for a season considered their cases perfectly hopeless. I have the pleasure to add, that I have had an opportunity, within these few days, of enquiring after their welfare, and am assured that not a vestige of disease remains.

It may be right and candid to confess, that the parties persevered in the diligent use of every means recommended, and submitted with readiness to every salutary discipline.

*Queen-street; August 6th,* 1819.

---

FOR THE LONDON MEDICAL AND PHYSICAL JOURNAL.

